# Recent Advances in Child and Adolescent Psychiatry

**DOI:** 10.3390/children9040489

**Published:** 2022-04-01

**Authors:** Matteo Chiappedi, Martina Maria Mensi

**Affiliations:** Child Neuropsychiatry Unit, IRCCS Mondino Foundation, 27100 Pavia, Italy; martina.mensi@mondino.it

The field of child and adolescent psychiatry is receiving growing attention, although a number of local differences still exist in terms of academic curricula, board certifications and even definitions of what is to be considered part of this field or not. An Italian study showed that approximately 1 out of 10 children showed significant psychopathological problems [1]; this study was conducted before the COVID-19 pandemic, and it is plausible (and seems to be confirmed both by clinical observation and literature findings [2]) that these figures are going to increase significantly in the next few months and years.

The main goal of this Special Issue is therefore to provide cutting-edge data regarding all aspects of child and adolescent psychiatry, including (but not limited to) the etiopathogenesis and presentation of the different disorders, treatment options and management of comorbidities. This is important because the peculiarities of the psychopathological manifestations in different age groups are increasingly recognized in classification systems, when a developmental perspective is applied [3].

Two papers in this Special Issue are case reports. Colizzi et al. provide a detailed description of a case of mosaic trisomy 20, analyzing the neuropsychiatric aspects in the light of a careful analysis of the existing literature [4]. Esposito et al. described the successful application of a stimulus discrimination training to reduce vocal stereotypies in an autistic child, offering an interesting background for future controlled studies [5].

Three more papers focused on different psychopathological sequelae of traumatic experiences. Calvano et al. provide evidence of the utility of a highly specialized approach in the form of an outpatient trauma clinic to start the therapeutic process and increase victims’ motivation towards a more prolonged intervention [6]. Forresi et al. described the effect of the exposure to an earthquake in a large sample of Italian families, showing the existing bidirectional correlation between parental and children internalizing symptoms and post-traumatic stress disorder [7]. Nehring et al. reported the utility of the Child Behavior Checklist Post-Traumatic Stress Disorder Scale and of an alternative scale (developed using a psychometrically guided item selection) as screening tools for post-traumatic stress disorder in a population of Syrian refugee children [8].

Two papers focused on the consequences of the COVID-19 pandemic. De Pasquale et al. studied the prevalence of online videogaming during the so-called “lockdown” in Italy, with anxiety (and especially state anxiety) being a relevant predictor of videogame use and addiction [9]. Baschenis et al. documented that children with dyslexia saw a reduction in their potential for reading evolution during school closure due to the COVID-19 pandemic, a fact that was coupled with greater social isolation and fewer worries about the pandemic and school closure [10].

The other papers testify the variety of subjects and approaches in child and adolescent psychiatry. Thun-Hohenstein et al. applied a naturalistic approach to assess the outcome of a group of adolescents consecutively admitted to an inpatient and day-clinic treatment [11]; they also showed that psychopathological severity at diagnosis and the relationship with the therapist were significantly related to the prognosis. Schwarz et al. provide initial findings regarding the understudied topic of the correlation between mental imagery and social pain, distinguishing its unique contribution not present in other anxiety-related disorders or in mood disorders [12]. Muratori et al. explored the utility of the Proposed Specifiers Conduct Disorder scale, and their findings support its use as a reliable, easy-to-use tool for measuring psychopathic traits in Italian children and young adolescents [13]. Brecht et al. studied a group of trans adolescents and evidenced a high level of psychopathological and especially internalizing problems, largely predicted by a reduced concordance between parental and subjects′ reports and by poor peer relations [14]. Iannattone et al. studied the role of alexithymia as a predictor of social withdrawal, especially when coupled with internalizing problems [15]. Last but not least, Bonati et al. provide the 10-year experience of the Lombardy ADHD Registry, offering many valuable insights concerning diagnosis and treatment emerging from this unmet experience [16], together with a description of unmet needs of these children and adolescents.

These contributions, with their variety in terms of explored topics and used methods, provide a mirror of the complexity of the topic of this Special Issue; hopefully, they will be thought- and action-stimulating reading for all those willing to pay comprehensive attention to children and adolescents.
